# Usefulness of gel immersion endoscopy for endoscopic resection in stomachs with residue

**DOI:** 10.1016/j.vgie.2025.03.040

**Published:** 2025-04-09

**Authors:** Hiroki Hayashi, Yuji Ino, Chihiro Iwashita, Mio Sakaguchi, Yoshimasa Miura, Edward J. Despott, Tomonori Yano, Hironori Yamamoto

**Affiliations:** 1 Department of Medicine, Division of Gastroenterology, Jichi Medical University, Tochigi, Japan; 2 Department of Gastroenterology and Hepatology, Mie University, Mie, Japan; 3 Department of Diagnostic Pathology, Jichi Medical University, Tochigi, Japan; 4 Division of Gastroenterology and Hepatology, Department of Medicine, Nihon University School of Medicine, Tokyo, Japan; 5 Royal Free Unit for Endoscopy, The Royal Free Hospital and UCL Institute for Liver and Digestive Health, London, United Kingdom; 6 Department of Endoscopic Research and International Education funded by FUJIFILM Medical Co, Ltd, Jichi Medical University, Tochigi, Japan

## Abstract

**Background and Aims:**

EMR and endoscopic submucosal dissection are widely used for treating intramucosal gastric neoplasms. However, securing a clear visual field in a stomach with residue is challenging. In this article, we present 2 cases in which tumors were endoscopically resected by securing the visual field using the gel immersion method in remnant stomachs after proximal gastrectomy with residue.

**Methods:**

The gel immersion method is a technique in which a transparent, viscous gel is injected into the lumen to secure the visual field. The viscous gel displaces blood and residue, allowing for a clear view. Therefore, we could perform efficient endoscopic procedures calmly. We also used the dedicated valve to add the gel while we inserted devices through its accessory channel.

**Results:**

In both cases, the gel immersion method successfully displaced food residue, providing a clear visual field and enabling precise mucosal incision and resection. In the first case, we achieved en bloc resection in a 71-year-old man with a 5-mm adenoma by using gel immersion EMR. In the second case, a 74-year-old man with a 12-mm intramucosal adenocarcinoma underwent gel immersion endoscopic submucosal dissection, which facilitated an accurate incision line and effective coagulation. Both patients had negative resection margins, and no adverse events were observed.

**Conclusions:**

The gel immersion method effectively improved visualization in a stomach with residue, enhancing the safety and precision of endoscopic resection.

## Introduction

**Figure 1:**
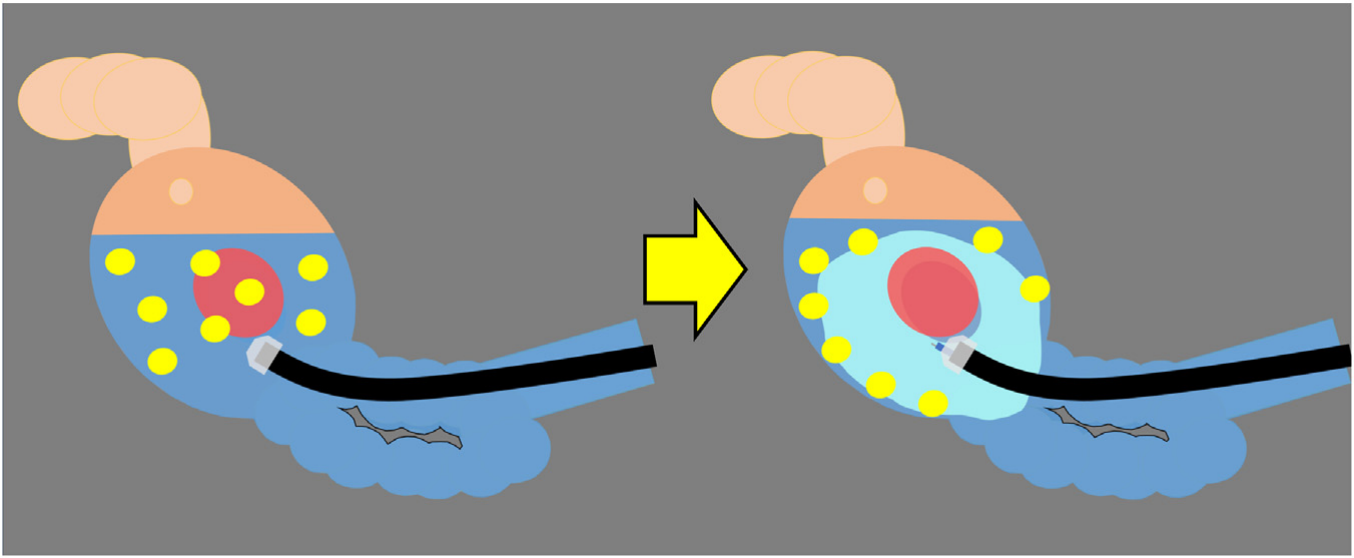
Residue can disrupt a clear visual field. The application of a viscous gel can clear food residue, and we can easily secure a therapeutic endoscopic view.

Intramucosal gastric neoplasms often are treated with EMR or endoscopic submucosal dissection (ESD). Securing a clear visual field is essential for identifying the resection site. However, keeping a satisfying therapeutic view in a remnant stomach, especially after proximal gastrectomy, often is difficult as the result of accumulated food residue, because the presence of the pyloric ring prevents food residue from being discharged. Extending the fasting period is one way to address residue, but it is not guaranteed. VISCOCLEAR (Otsuka Pharmaceutical Factory, Tokushima, Japan) is an electrolyte-free gel, ensuring no current leakage during energization, even in devices with a large contact area.^1^ Therefore, mucosal incision, dissection, and coagulation can be performed effectively^2^ in the space filled with the dedicated gel. This gel can clear food residue away from the resection site as the result of its viscosity; therefore, we can easily secure a clear therapeutic endoscopic view (Fig. 1). We describe 2 cases of gastric neoplasms resected endoscopically with the dedicated gel in such remnant stomachs (Video 1, available online at www.videogie.org).

## Gel immersion method

**Figure 2:**
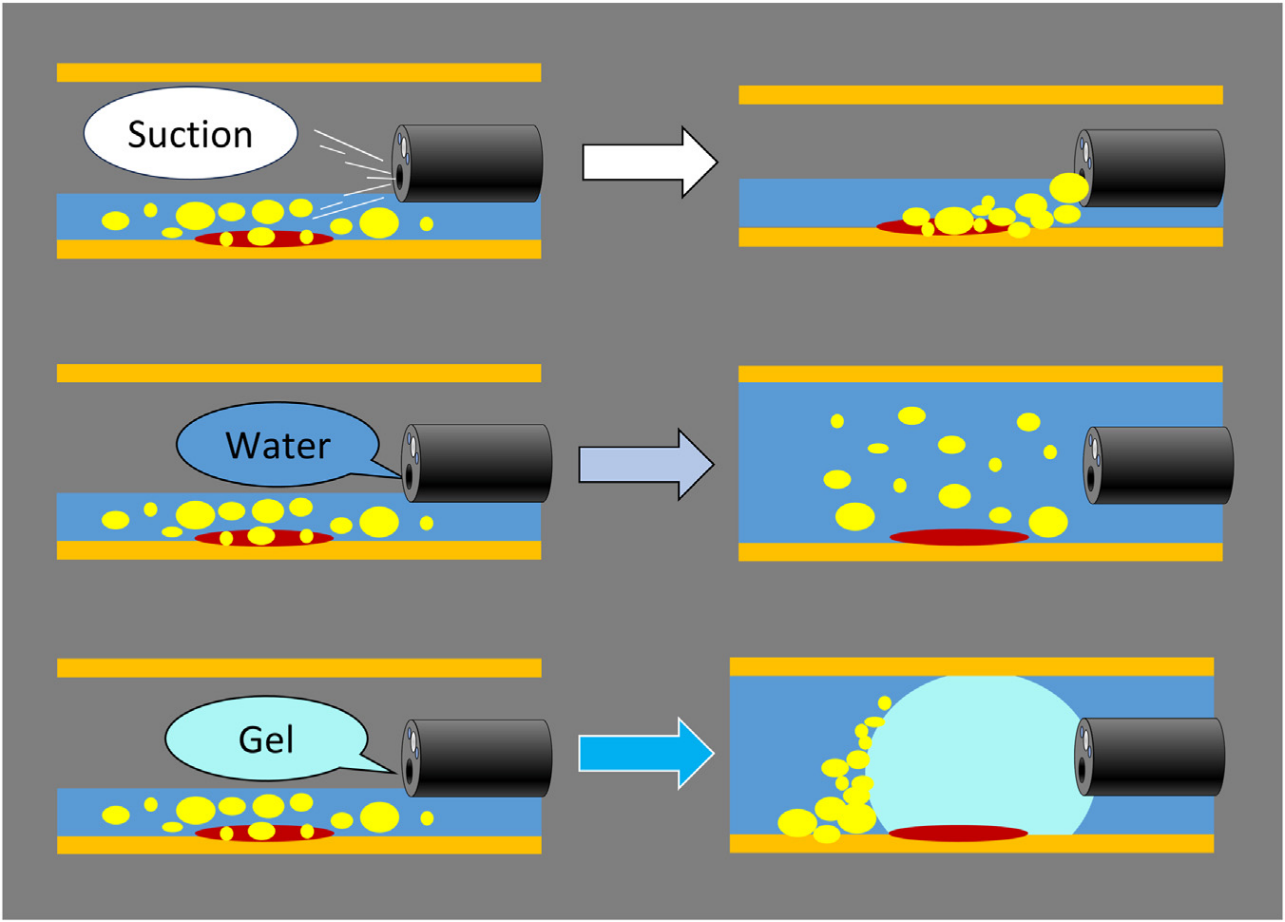
Residue often clogs the suction port. Floating residue disturbs the observation of gastric mucosa underwater. The application of a viscous gel displaces the residue and facilitates clear visualization of gastric mucosa.

We injected the gel into the remnant stomach at first. The viscous gel displaced the residue, facilitating mucosal visualization^3^^,^^4^ (Fig. 2). Therefore, we could perform efficient endoscopic procedures calmly. We also used BioShield irrigator (US Endoscopy, Mentor, Ohio, USA) to add the gel while we inserted devices through its accessory channel.

## Case 1

**Figure 3:**
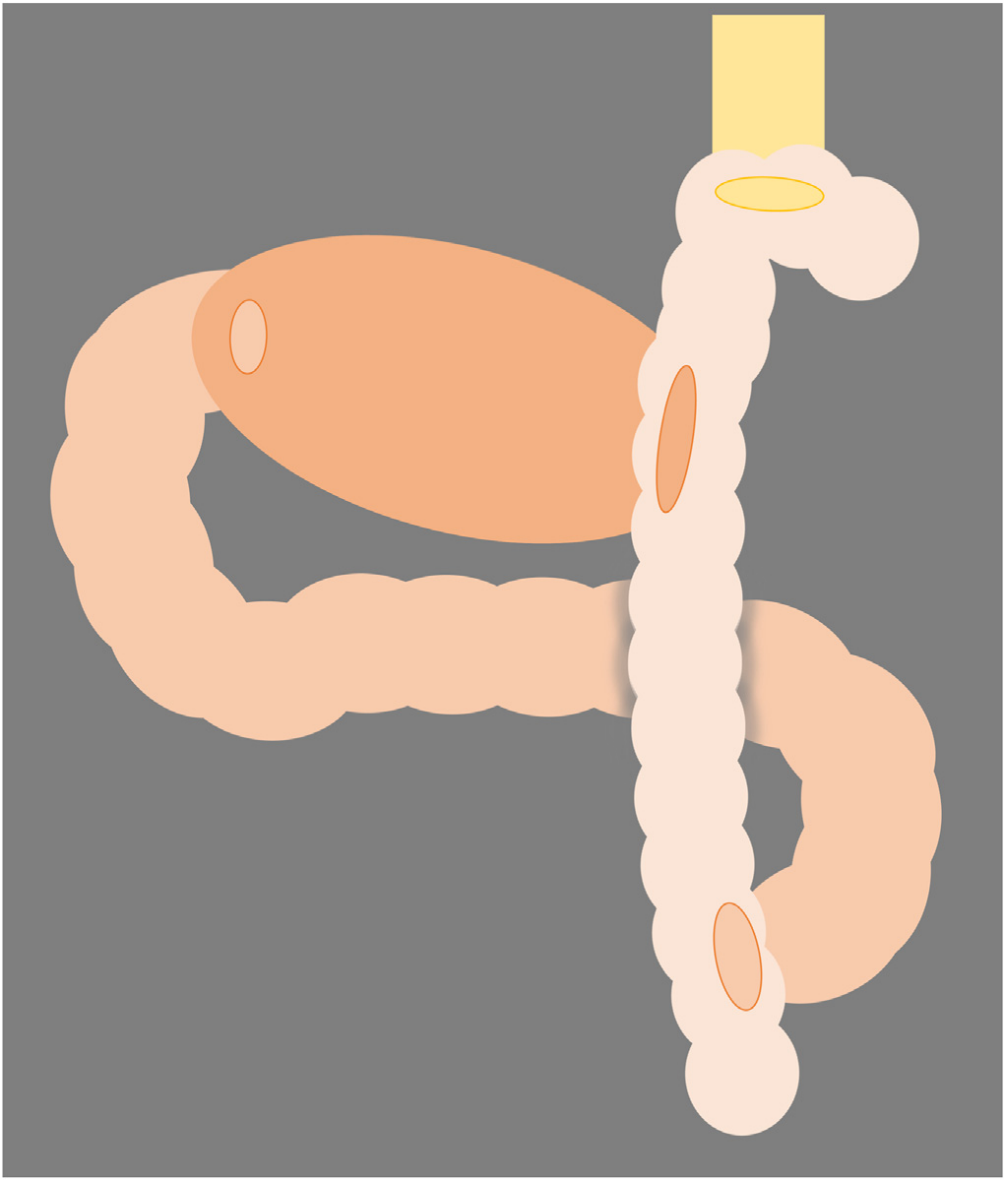
The double-tract reconstruction involves esophagojejunostomy, gastrojejunostomy, and jejunojejunostomy. This method has the advantage of a lower incidence of reflux esophagitis.

**Figure 4:**
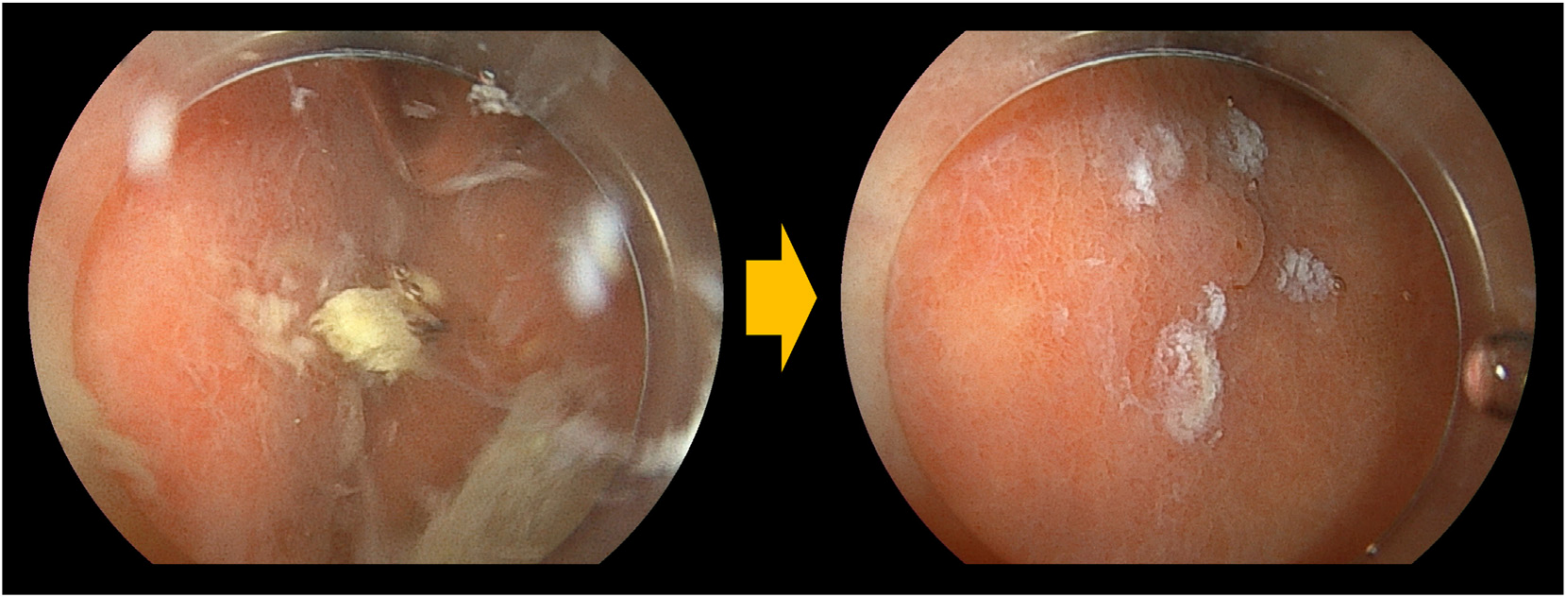
The floating residue impaired the visual field underwater; however, the gel effectively displaced the residue, dramatically enhancing visualization.

**Figure 5:**
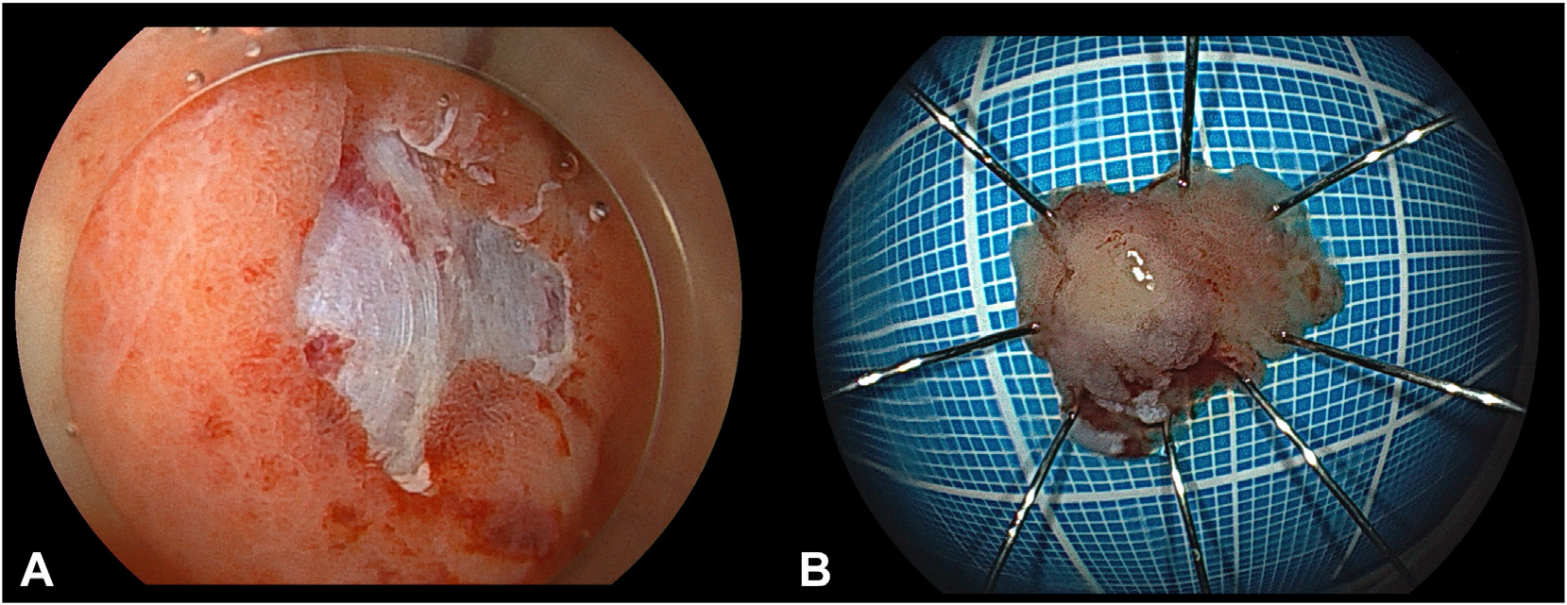
**A,** Endoscopic imaging confirmed no residual tumor, and (**B**) the resected specimen contained the entire tumor.

**Figure 6:**
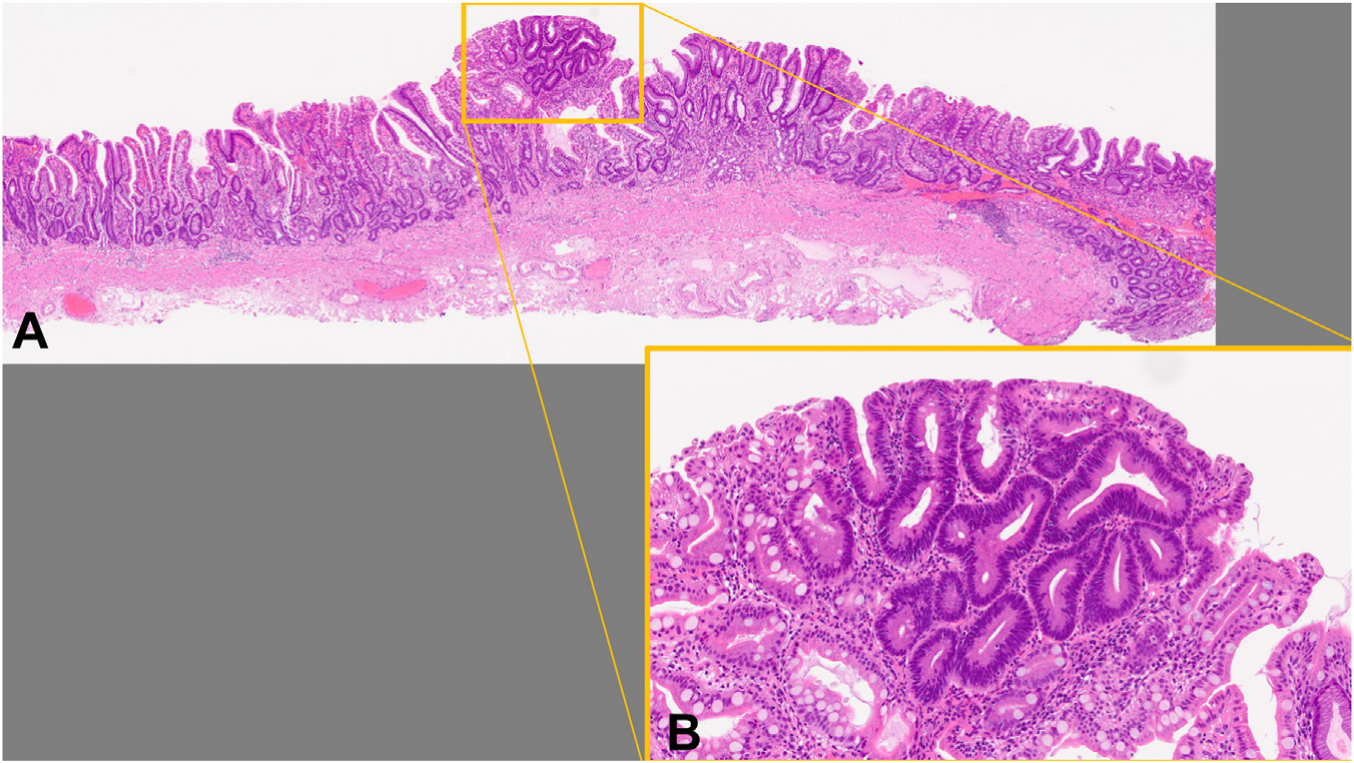
Pathologic findings showed tubular adenoma, and resected margins were negative: (**A**) hematoxylin eosin stain, orig. mag ×40, and (**B**) hematoxylin eosin stain, orig. mag. ×100.

A 71-year-old man underwent proximal gastrectomy and double-tract reconstruction (Fig. 3)^5^ for gastric cancer 8 years previously. Six years later, EGD revealed a 5-mm slightly elevated lesion in the remnant stomach. We planned underwater EMR to prevent slippage of the snare and to allow for reattempting the procedure if necessary.^6^ However, a great amount of residue was disturbed, keeping us from visualizing of the lesion entirely. Because adequate visualization for EMR could not be achieved under either gas insufflation or underwater conditions, gel immersion was adopted.^7^ The gel displaced any residue, dramatically enhancing the visual field (Fig. 4). We could snare the lesion, confirming the markings and achieved en bloc resection (Fig. 5). Pathologic findings showed tubular adenoma, and resected margins were negative (Fig. 6).

## Case 2

**Figure 7:**
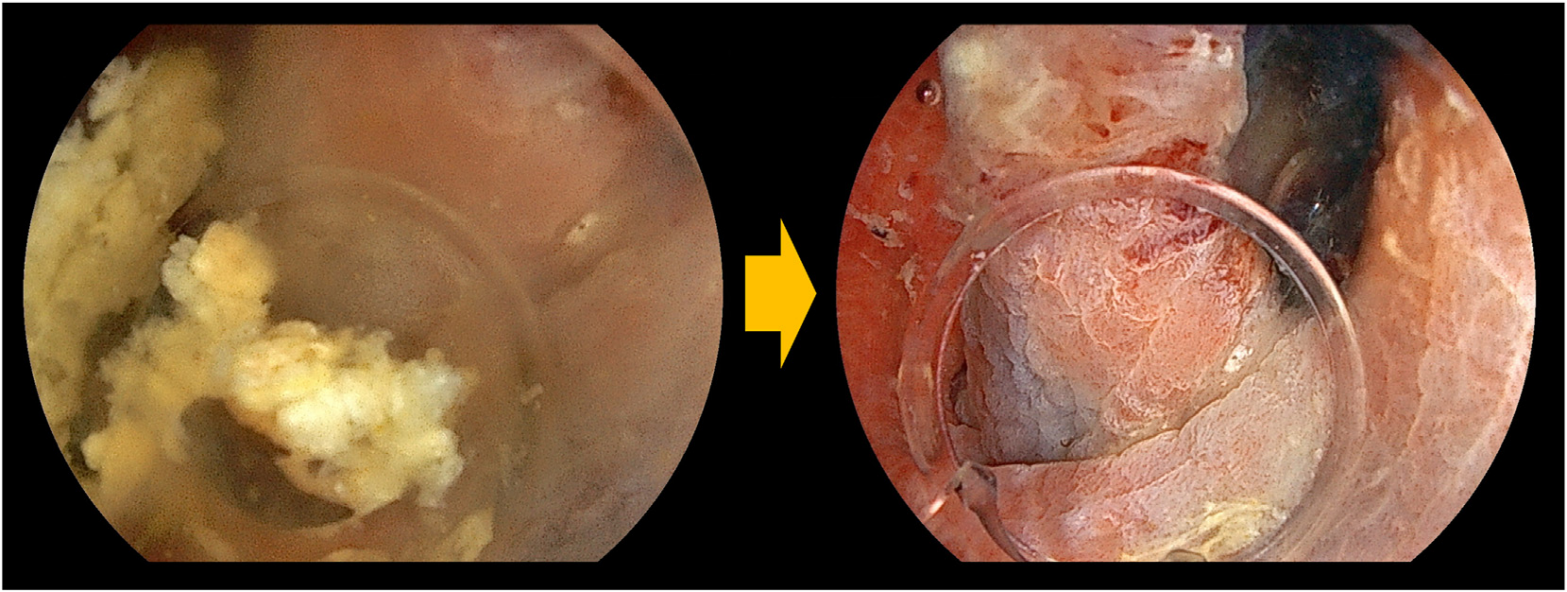
Underwater, the visual field was poor as the result of floating residue, obscuring the incision line; however, the application of the gel effectively displaced the residue, thereby improving visualization.

**Figure 8:**
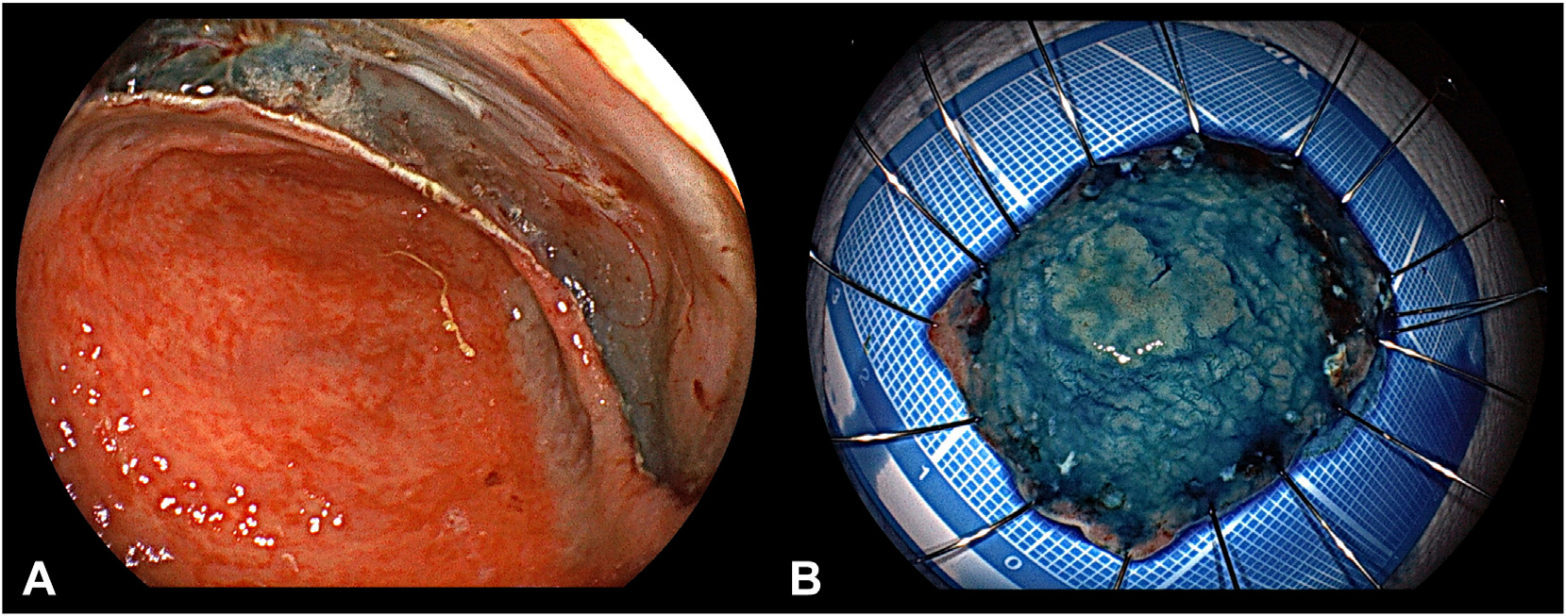
**A,** Endoscopic imaging confirmed the absence of residual tumor, and (**B**) the resected specimen contained the entire tumor.

**Figure 9:**
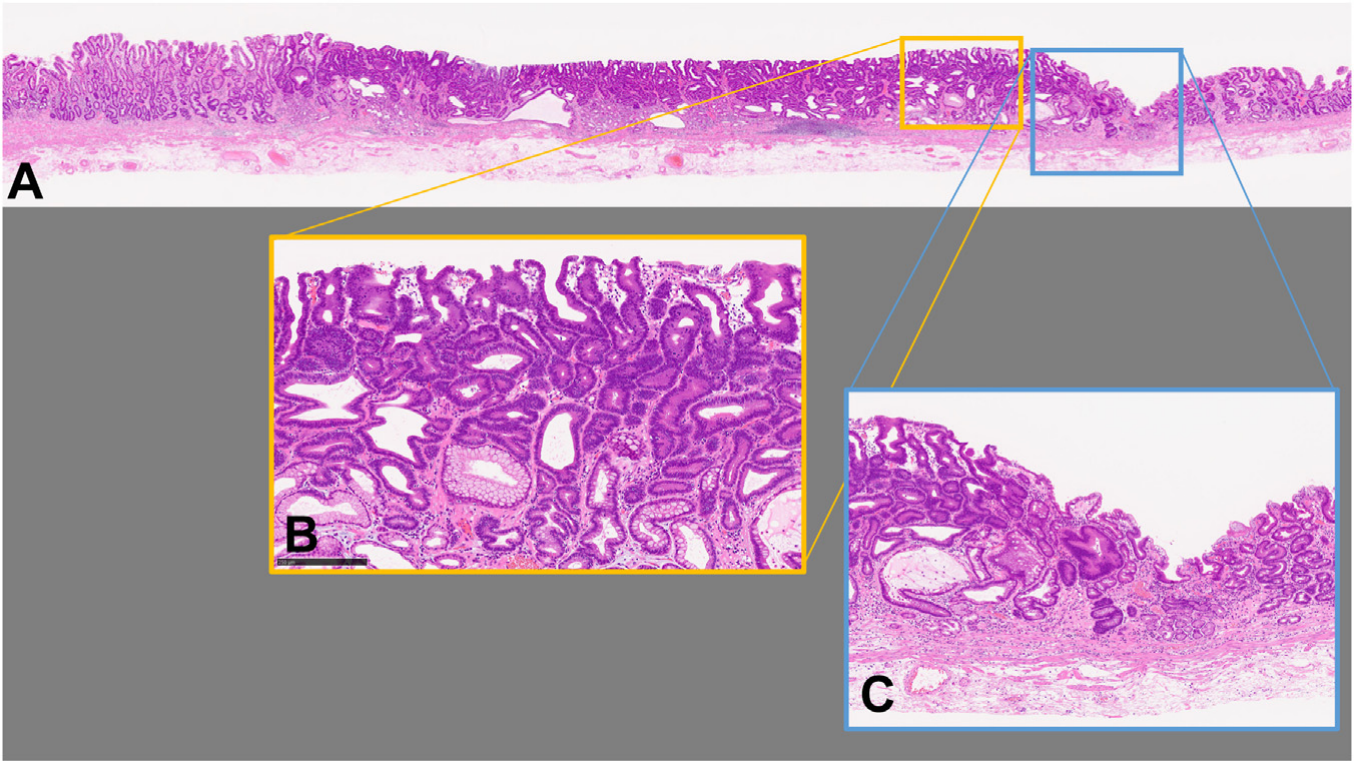
Pathologic findings showed well-differentiated intramucosal adenocarcinoma and resected margins were negative. **A,** Hematoxylin eosin stain, orig. mag. ×4; (**B**) hematoxylin eosin stain, orig. mag. ×40; (**C**) hematoxylin eosin stain, orig. mag. ×100).

A 74-year-old man underwent proximal gastrectomy and jejunal sac reconstruction 21 years previously for early-stage cancer of gastric cardia. Recent follow-up EGD revealed a 12-mm 0-IIa lesion in the remnant stomach. Endoscopic findings suggested intramucosal carcinoma, and ESD was performed. ESD proceeded smoothly on the proximal side with gas insufflation. However, on the greater curvature side, the incision line could not be determined because of accumulated food residue. Saline immersion worsened visibility. Consequently, gel immersion ESD was attempted using the electrolyte-free gel. The gel displaced the residue, significantly improving the visual field (Fig. 7). The incision line became clear, and we proceeded with successful en bloc resection (Fig. 8). Gel immersion also facilitated effective coagulation of any bleeding vessels through the provision of a clear view. Pathologic findings showed well-differentiated intramucosal adenocarcinoma, and resected margins were negative (Fig. 9).

In both cases, the gel worked as expected by displacing any residue and providing a clear field of view. This effect can be expected regardless of surgical history. Furthermore, coagulation hemostasis with hemostatic forceps was also successfully achieved within the gel-filled dissection space, as previously reported.^1^^,^^2^ This gel immersion method requires less than 1 minute from preparation to filling the field of view, provided the lumen is adequately degassed. It is a useful method that allows us to avoid postponing endoscopic procedures.

## Conclusions

The gel immersion method improved the visual field in a remnant stomach after proximal gastrectomy and facilitated endoscopic therapy of gastric neoplasms in this scenario.

## Patient Consent

The patients in this article have given written informed consent to publication of the case details.

## Disclosures

E. J. Despott reports academic grants and speaker honoraria from Fujifilm. T. Yano reports a patent for the dedicated gel, is an advisor for the company, and receives speaker honoraria from Otsuka Pharmaceutical Factory and speaker honoraria from Fujifilm. H. Yamamoto reports being a consultant for Fujifilm. The other authors have no financial disclosures to declare.

## Figures and Tables

**Video still:**
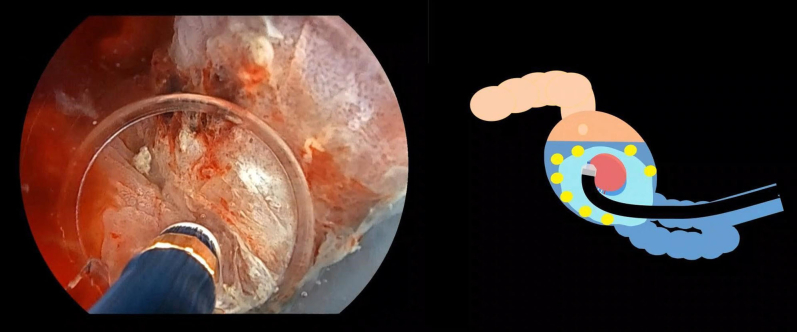


## Supplementary data

Video 1 Successful resection of gastric neoplasms in the remnant stomach with residues using the gel immersion method.

## References

[1] Yano T., Ohata A., Hiraki Y. (2021). Development of a gel dedicated to gel immersion endoscopy. Endosc Int Open.

[2] Khurelbaatar T., Miura Y., Yano T. (2022). Electrolyte-free gel immersion endoscopic submucosal dissection of gastric lesions. Endoscopy.

[3] Yano T., Nemoto D., Ono K. (2016). Gel immersion endoscopy: a novel method to secure the visual field during endoscopy in bleeding patients (with videos). Gastrointest Endosc.

[4] Yano T., Takezawa T., Hashimoto K. (2021). Gel immersion endoscopy: innovation in securing the visual field—clinical experience with 265 consecutive procedures. Endosc Int Open.

[5] Kim D.J., Kim W. (2016). Laparoscopy-assisted proximal gastrectomy with double tract anastomosis is beneficial for vitamin B12 and iron absorption. Anticancer Res.

[6] Hashimoto K., Ino Y., Ishii H. (2024). Gel immersion endoscopic mucosal resection for small gastric neoplastic lesions: a pilot study. DEN Open.

[7] Kimura H., Yamamoto Y., Yabuuchi Y. (2024). Gel immersion endoscopic mucosal resection for early gastric neoplasms: a multicenter case series study. Endosc Int Open.

